# The Diverse Roles of the MEF2 Transcription Factor Family in Tumor Progression and Emerging Therapeutic Opportunities

**DOI:** 10.3390/biomedicines14081692

**Published:** 2026-07-28

**Authors:** Yanyan Chen, Jingni Zhu, Jinghang Qian, Sheng Li, Liu Yang

**Affiliations:** 1 Department of Oncology, The Affiliated Cancer Hospital of Nanjing Medical University & Jiangsu Cancer Hospital & Jiangsu Institute of Cancer Research, Nanjing 210009, China; cyybhfq@163.com (Y.C.); nini198665@163.com (J.Z.); 2 Department of Colorectal Surgery, The Affiliated Cancer Hospital of Nanjing Medical University & Jiangsu Cancer Hospital & Jiangsu Institute of Cancer Research, Nanjing 210009, China; q1222jh@163.com; 3 Jiangsu Key Laboratory of Innovative Cancer Diagnosis & Therapeutics, Nanjing 210000, China

**Keywords:** MEF2 family, transcriptional regulation, malignant hallmark, targeted therapy

## Abstract

The myocyte enhancer factor 2 (MEF2) transcription factor family plays crucial roles in differentiation, lineage specification, stress responses, and tissue homeostasis. Recent investigations have shown that the dysregulation of MEF2A, MEF2B, MEF2C, and MEF2D is associated with tumorigenesis, tumor progression, and adverse clinicopathological features in several cancers. MEF2B has a particularly important role in B-cell malignancies, where recurrent mutations deregulate BCL6 and promote lymphoma progression. MEF2A, MEF2C, and MEF2D also regulate malignant phenotypes, including proliferation, migration, invasion, apoptosis, drug resistance, angiogenesis, inflammation, and immune evasion, by the mechanism of regulating cell-cycle regulators, apoptosis-related genes, EMT-related genes, and other transcriptional programs. This review summarizes the mechanisms by which MEF2 family members contribute to tumor initiation and progression, with added emphasis on *MEF2B* mutations and *MEF2D* fusions. We also discuss clinical associations with overall survival and recurrence in solid tumors and hematologic malignancies. Because MEF2 proteins are transcription factors with broad physiological functions, we evaluate therapeutic strategies: RNA interference, genetic perturbation, small molecules that alter MEF2-dependent transcription, and PROTAC or oligonucleotide-PROTAC. These platforms remain promising but require MEF2-specific validation, tumor-selective delivery, and careful toxicity assessment.

## 1. Introduction

The myocyte-specific enhancer factor 2 (MEF2) protein, including four members, MEF2A, MEF2B, MEF2C and MEF2D, was initially identified by researchers in 1989 as the nuclear protein that interacts with the enhancer region of the muscle creatine kinase gene (MCK) during muscle differentiation [1]. The MEF2 genes are widely expressed in many species of eukaryotes, including homo sapiens [2], acting as master regulators of target genes [3]. MEF2 families play crucial roles in the essential progression of differentiation, cell stemness, proliferation and in various adaptive responses in different tissues. MEF2A and MEF2D are widely expressed across various human tissues, with particularly high enrichment in the brain, heart, and skeletal muscle [4]. MEF2C is predominantly localized to the central nervous system, the cardiovascular system (including endothelial cells) [2], and specific hematopoietic lineages, whereas MEF2B expression is largely confined to the brain, testes, and germinal center B cells [5]. In addition, MEF2C is absolutely essential for early embryonic development; global *Mef2c* knockout mice are embryonic lethal at day 9.5 due to severe cardiovascular defects, including the failure of cardiac looping and the absence of a right ventricle [6].

Transcription factor imbalance may be one of the causes of tumorigenesis. For example, NEPC-enriched TFs induce the progression of prostate adenocarcinoma (PRAD) to neuroendocrine prostate cancer (NEPC) [7]. In recent years, increasing evidence has confirmed the molecular mechanisms and pathways related to MEF2 families and the differences in the members in different solid tumors, lymphomas and leukemias. In some cases, the function of MEF2s in cancer cannot be clearly classified, although most studies indicate that MEF2s act as oncogenes. This review surveys the molecules, signal activation, cell and animal models about the MEF2 deregulation reported in the literature before and emphatically updates the latest 5-year tumor studies. We then discuss the role of MEF2 proteins in malignant processes, focusing on the recent significance of MEF2 proteins in cancer proliferation, migration, metastasis, drug resistance, and angiogenesis. At the same time, we summarized the research results of MEF2 on the prognosis and pathological characteristics of clinical patients with various blood tumors and solid tumors, illustrating the mechanism of context-dependent transcription factors in tumor progression.

The review will be helpful in finding the potential intervention targets for new therapeutic methods in the future by unraveling and summarizing the regulatory mechanism of the MEF2 and its clinical value. There has been considerable research conducted on the relationship between MEF2 and the clinical prognosis of tumor patients and its predictive value. Referring to other transcription factor intervention methods, although there are currently no preclinical drugs directly targeting the MEF2 transcription factor, they still have unlimited potential for the future. However, translating MEF2 biology into therapy remains challenging because MEF2 proteins are DNA-binding transcription factors that share conserved MADS/MEF2 domains and interact with broadly used co-regulators. Therefore, this review distinguishes established preclinical data from more speculative therapeutic concepts and highlights the need for isoform-selective or tumor-selective strategies.

## 2. MEF2 Family Proteins

**Figure 1:**
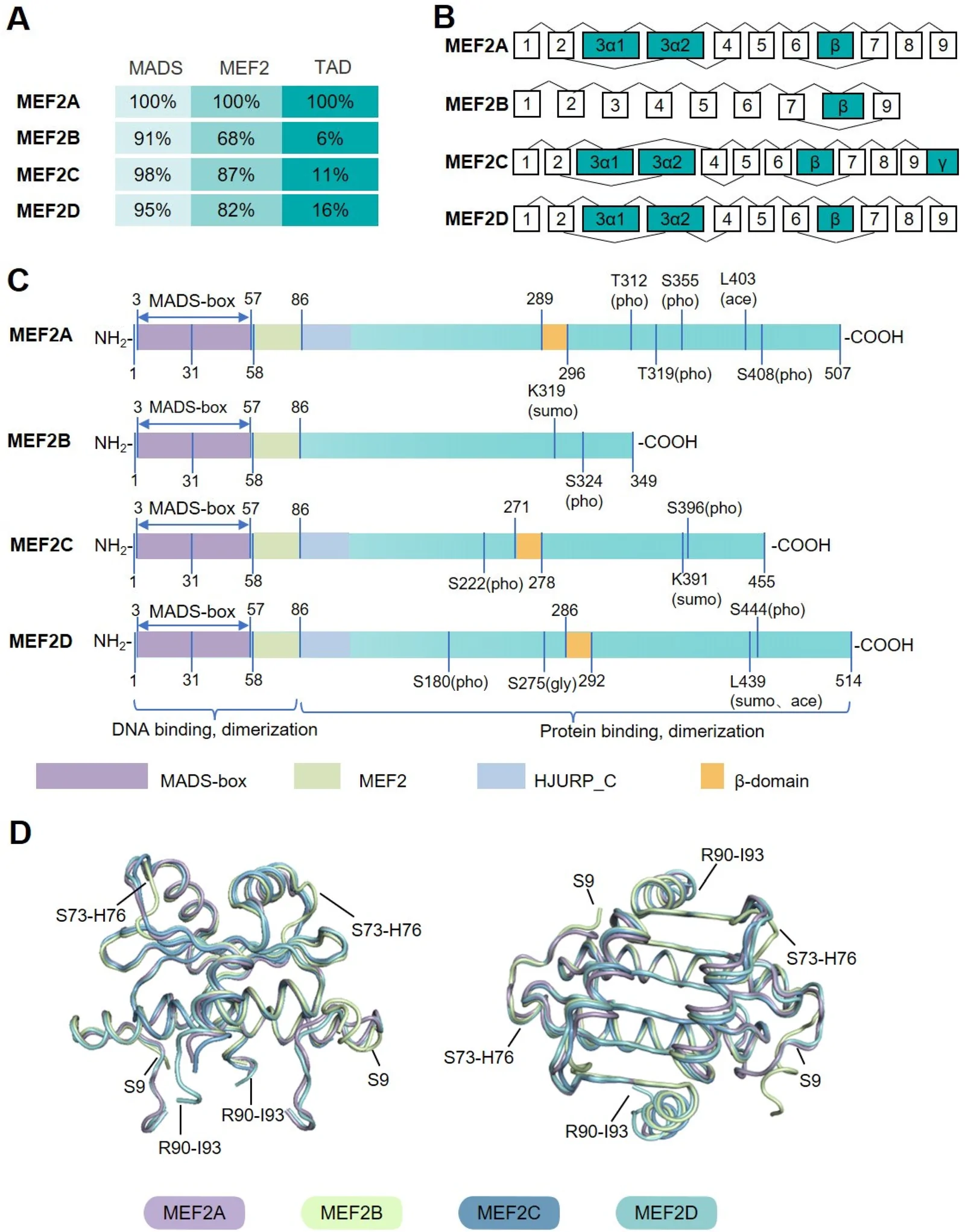
(**A**) Gene sequence homology of the MEF2 families across different factors. The percentage of sequence identity is calculated relative to human MEF2A, served as the reference 100%. (**B**) MEF2s have similar gene structures and alternative splicing patterns among coding exons. There are nine common exons in the coding region of MEF2A, MEF2C and MEF2D, among which two alternatively spliced third exons 3α1 and 3α2 exist mutually exclusive in the mature RNA transcript. In contrast, MEF2B is the structurally simplest of the MEF2 proteins, the splicing of which mainly brings two isoforms, respectively made up of 8st and 9st exons. (**C**) The structure and common modification sites of the MEF2 protein share a similar N-terminus, including the highly conserved MADS-box domain and the adjacent MEF2 domain. The former consists of 55 amino acids, and the latter has 29 amino acids, respectively. In addition, β exon is translated into TAD. The structure of the C-terminal transactivation domain of MEF2 protein is diverse and contains multiple amino acids with the potential to be phosphorylated, SUMOylated, and glycosylated. (**D**) Protein superimposition of aligned MEF2A (PDB ID: 3KOV), MEF2B (PDB ID: 6C9L), MEF2C (PDB ID: 5F28) and MEF2D (PDB ID: 8Q9N) are shown in two orientations (90° rotation). In the conserved N-terminal domain, the sequences with the most significant structural variation are S9, S73-H76 and R90-I93 of the MEF2B molecule, where the loops have a high degree of variability.

In humans, the *MEF2A*, *MEF2B*, *MEF2C*, and *MEF2D* genes are found at distinct chromosomal locations: 15q26, 19p12, 5q14, and 1q12-q23, respectively. Despite their non-proximity in terms of chromosomal locations, these genes exhibit a high degree of sequence similarity [8]. From the perspective of gene sequence and homology, the MEF2B gene appears to be more distantly related to the other three *MEF2* genes and is presumed to have the most ancient origin [9] (Figure 1A). Furthermore, after differential alternative splicing events, there are significant differences between the mature transcripts of different MEF2 (Figure 1B).

In terms of protein structure and superimposition, the MEF2 family belongs to the type II MADS domain-containing proteins (Mini-chromosome Maintenance 1, agamous, deficient and serum response factor), which contain a conserved MADS domain and MEF2 domain and a less conserved transactivation domain (TAD) [10] (Figure 1C). The MADS domain is involved in non-specific binding to DNA and protein partners, while the MEF2 domain interacts with specific DNA sequences. The protein molecular structures of different MEF2 members are relatively conserved, but the similarity of MEF2B is lower (Figure 1D). However, different or similar expression patterns are often observed among the four family members depending on the specific cellular context, different tissues, lineages, and differentiation stages [10,11].

### 2.1. Cancer-Associated MEF2 Gene Mutations

Disease-associated MEF2 alterations are most clearly established in hematologic malignancies. MEF2B mutations occur recurrently in follicular lymphoma and diffuse large B-cell lymphoma and deregulate BCL6 expression, supporting a driver role in B-cell lymphomagenesis [6]. MEF2D fusions, including MEF2D-BCL9 and related rearrangements, define high-risk subsets of B-cell precursor acute lymphoblastic leukemia and are associated with adverse clinical features [12,13,14]. MEF2C dysregulation has also been reported in immature T-cell acute lymphoblastic leukemia and acute myeloid leukemia [15,16]. These genetic and transcriptional alterations justify discussing MEF2 family members not only as deregulated expression markers but also as cancer-associated mutant or fusion genes.

### 2.2. MEF2A

As an intracellular protein, Myocyte enhancer factor 2A (MEF2A) acts in both the cytoplasm and nucleus. Protein domains for MEF2A include MADS_MEF2-like, TF_MADS box, TF_MADS box_sf and HJURP_C (Holliday junction recognition protein C-terminal) [17] (Figure 1C). MEF2A, acting as a homo- or heterodimer [18,19], is a direct DNA-binding regulator that activates a large number of transcriptional upregulation of muscle-specific, growth factor-induced and stress-inducible genes [20]. Both interactor and post-translational modifications for MEF2A can influence the transcription activity (Figure 1C,D). Phosphorylation on Ser-408 is the main factor that shows vital functions on MEF2A transcriptional activation [11,21]. MEF2A is highly expressed in gastric cancer and phosphorylated by p38, which is regarded as activated and promotes glycolysis by transcribing GLUT4 [22].

### 2.3. MEF2B

**Figure 2:**
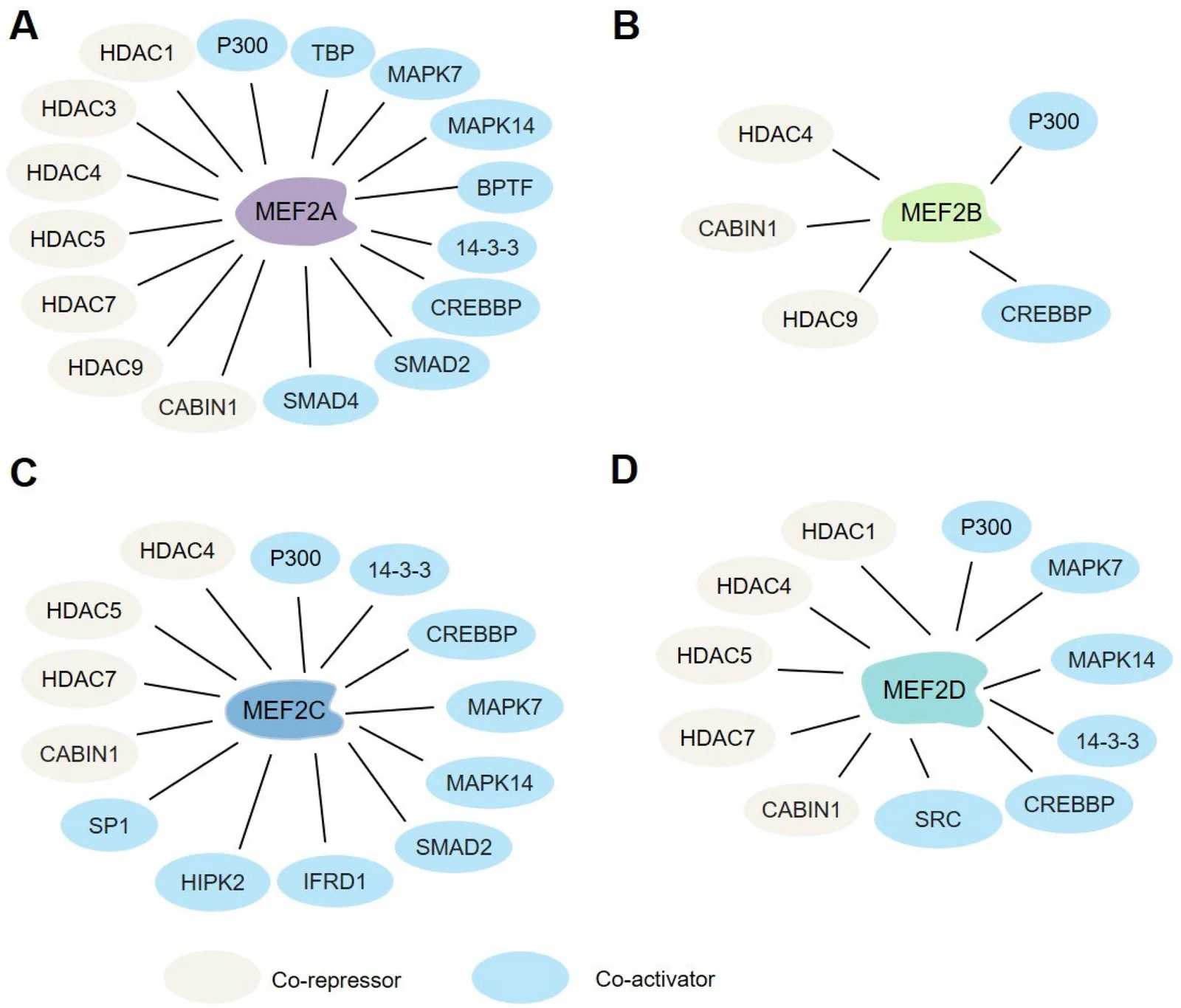
Interacting proteins of the different MEF2 transcription factors with enhancing or inhibiting the transcriptional activity. These interactors are classified into the co-activator that enhances or the co-repressor that reduces transcriptional activity of (**A**) MEF2A, (**B**) MEF2B, (**C**) MEF2C and (**D**) MEF2D.

Myocyte enhancer factor 2B (MEF2B) can be spliced and translated into several isoforms with a missing HJURP_C region, causing a shorter amino acid sequence compared with other family members (Figure 1C,D). The molecule interacts with the dimer composition of the DNA-binding complex by specifically binding to the 5′-YTA[AT](4)TAR-3′ elements in numerous muscle-specific genes as a transcriptional activator [21]. At the same time, dimer formation with interacting molecules also affects the activity of transcription factors. MEF2B protein interacts with members of the MyoD family of transcriptional activators and forms dimers to initiate the transcription of downstream genes [22]. MEF2B protein also binds to TWIST to inhibit myogenesis, and is negatively regulated by MITR in association with HDAC1 [23] (Figure 2B). Furthermore, MEF2B mutations commonly occur in approximately 15% of follicular lymphoma cases. These results provide strong evidence that MEF2B alterations are involved in the pathogenesis of these lymphoma subtypes and highlight their potential as biomarkers in lymphoid malignancies [6,24].

### 2.4. MEF2C

Similarly, Myocyte Enhancer Factor 2C (MEF2C) has both DNA-binding and trans-activating activities [3,23]. There are a total of six domains from N-terminal to C-terminal in MEF2C: MADS, MEF2, HJURP-C, TAD1 and 2, and NLS, with some alternative donor/acceptor splicing sites [24,25] (Figure 1C). Deletions and mutations in these loci encoding critical domains have been linked to diseases such as stereotypical movements, epilepsy, cognitive impairment and brain malformations [26,27]. This protein is implicated in maintaining the differentiated state of muscle cells [28], myogenesis [29] and cardiac morphogenesis, and neurogenesis and the development of cortical architecture [30]. In terms of tumor cell differentiation, some evidence shows that MEF2Cα2 improves differentiation in rhabdomyosarcoma (RMS) cells, while the expression of the MEF2Cα1 exon inhibits differentiation, demonstrating distinct roles of different MEF2C isoforms in tumor progression [31].

### 2.5. MEF2D

The sequence length of Myocyte Enhancer Factor 2D (MEF2D) protein is about 500 Aas—the longest protein in the MEF2 family, containing the HJURP_C region domain such as MEF2A and MEF2C (Figure 1C,D). Several key sites in the TAD of MEF2D can interact with other transcription factors or coactivator proteins to regulate the transcriptional activity of the gene. MEF2D can interact with members of the histone deacetylase (HDAC) family (Figure 2A), which enables MEF2D to participate in histone deacetylated modifications and chromatin remodeling, thereby affecting the transcriptional activity of genes [32,33]. This factor mediates cellular functions not only in muscle development [4] but also in neuronal differentiation and survival [34]. This molecule plays multiple roles in the regulation of the muscle-specific and/or growth factor-associated transcription by the canonical p38 MAPK signaling [35]. At the same time, as an important regulator of the pre-metastasis microenvironment, MEF2D plays an important role in promoting hepatocellular carcinoma (HCC) and metastasis [36].

## 3. The Factors That Influence MEF2 Transcription Activity

The MEF2 protein is a key regulator of transcriptional activity in the nucleus. Previous studies have shown that MEF2s serve as scaffolding proteins within the cell nucleus and interact with intracellular proteins and genes. There are relatively few studies on the MEF2s that do not depend on the transcriptional role. The capacity for transcription is influenced by the following factors: the folding structures and domains of MEF2 proteins, the co-regulators that interact with the transcription factors, and the post-translational modification of key sites (Figure 2A–D).

### 3.1. The Folding Structure and Domains That Are Related to MEF2s Transcriptional Activity

The MADS-box, MEF2 and C-terminal are the main domains of MEF2 members (Figure 1C). It is worth noting that the specific DNA sequence and structure recognition may vary among different MEF2 isoforms, different cell types, and different contexts [37]. The conserved MADS-box domain always facilitates mainly binding with the CC[A/T]6GG motif in DNA sequence [38]. The MEF2 domains can also interact with DNA structures that have bends or deformations. Elevated DNA bending has also been linked to the high-affinity binding of the MADS-box domain [38].

The MEF2 domain exhibits a significant level of conservation among different MEF2 families, and its specific conservative sequence is typically represented as 5′-T(A/T)A(A/T)T(A/T)T-3′. This specific sequence is commonly found in the upstream region of the MEF2 binding site, and it collaborates with the MADS domain to form a complex that binds to DNA [39]. Furthermore, despite the high conservation of the N-terminus, each MEF2 exhibits some differences in preference for DNA binding since the C-terminal regions of MEF2 proteins are highly divergent [39] (Figure 1C,D). Typically, the interaction between the well-characterized MEF2 chaperone and the MADS and MEF2 domains is facilitated by the hydrophobic pocket formed by Leu66, Tyr69, and Thr70. And the helix H2 plays a crucial role in mediating this interaction [17] (Figure 1D). These MEF2 partners possess a consensus sequence, referred to as the MEF2 binding sequence, which fits snugly into this groove [17]. Although neither the MADS box nor the MEF2 domain has its own transcriptional site activity, the MEF2 domain regulates DNA binding affinity and interactions with cofactors; other MEF2 proteins are critical for MEF2 transcriptional activity [40].

The C-terminal transactivation domain varies significantly among different members of the MEF2 protein family due to gene sequence differences and some tissue-specific alternative splicing [39,41] (Figure 1B,C). The diverse C-terminal transactivation domains of MEF2 proteins interact with different transcriptional co-regulators in various biological contexts. These interactions involve co-activators like the widely recognized P300 and CBP (CREB-binding protein) acetyltransferases [40,42], as well as co-repressors such as the classic class II histone deacetylases and SMRT (silencing mediator of retinoic acid and thyroid hormone receptor) [43,44] (Figure 2A–D). The MADS and MEF2 domains and TAD constitute pivotal pillars in the transcriptional activity of MEFs, ultimately influencing the affinity of DNA binding and the rate of transcription initiation.

### 3.2. The Interactors Related to MEF2s Transcriptional Activity

There are many types of proteins that bind to the MEF2 transcription factor, including histone- and non-histone-modifying enzymes, other transcription factors, transcriptional coactivators, and signal transduction proteins [45]. The interaction of these proteins with MEF2 can affect the transcriptional regulatory function of MEF2, regulating gene expression and cell function (Figure 2A–D). The protein sequence XX(V/T/I)(K/R)XZ(L/F)ZXX(V/I/L)XXX is considered a consensus partner for binding to MEF2 in previous reports [17].

At times, different types of co-activators and repressors may compete to interact with the same region of MEF2. This mechanism is also one of the important mechanisms affecting MEF2 transcriptional activity. The overall transcriptional activity of MEF2 is tightly controlled by the mutually exclusive effects of HDAC or p300/CBP, a common set of interacting partners, by which MEF2 regulates the activation or repression of a regulatory element [46]. Furthermore, MEF2 proteins have been observed to interact with both histone acetyltransferases (HATs) and histone deacetylases (HDACs), which play crucial roles in regulating the epigenetic status of the regulatory region [47]. MEF2s associate with the chromatin remodeling proteins, like class IIa HDACs, such as HDAC4, -5, -7 and -9, by MEF2 domain in the resting state and co-recruit HP-1 (heterochromatin protein 1), CtBP (C-terminal binding protein), and class I HDACs (histone deacetylases) to specific loci of the genome, which leads to the inhibition of target gene transcription [47,48]. The transcription will be activated by the upstream Ca^2+^-dependent mechanisms that lead to MEF2s—as scaffold proteins—interacting with Ca^2+^-sensitive transcription factors, such as NFAT (nuclear factor of activated T cells) or CREB (cAMP response element-binding protein), and facilitating the recruitment of p300/CBP (CREB-binding protein) to promoters. This recruitment enables transcriptional activation of downstream genes [33,46,49,50]. At the same time, these HDACs and acetylases also participate in epigenetic regulation by recruiting chromatin remodeling complexes to acetylated histones, and in this way participate in the activation of MEF2-dependent transcriptional regulation [51]. However, when HDACs are released into the cytoplasm, and MEF2 in the nucleus can interact with other proteins for transcriptional activation, acetylation becomes a sign of transcriptional repression [52].

Apart from enzymes, intranuclear proteins, including other transcription factors, also interact with MEF proteins and modulate their transcription activity as co-regulators of transcription. In B-cells, for instance, the stabilized β-catenin protein is translocated into the nucleus, where it forms complexes with MEF2 proteins through interactions [53,54]. Transcription elongation factors may be involved in the regulation of MEF2 transcriptional activity. For example, the positive transcription elongation factor b (P-TEFb) interacts with MEF2, which then leads to the hyperphosphorylation of the C-terminal region of RNA polymerase II, effectively enhancing the speed of transcription, which relies on the formation of intricate dimerization complexes of MADS-MEF2 domains [47,55].

The modification of key sites of transcription factors may change the ability of MEF2s to bind to other proteins and thus affect the transcriptional activity of MEF2s. Consequently, mutations in specific sites of MEF2s can result in positive or negative effects on the transcriptional activities of MEF2s, and the outcome is contingent upon the stability of the respective complexes within the epigenetic context and specific cell lineage. Consequently, mutations in specific MEF2 regions may have activating or repressive effects depending on the epigenetic context, lineage, and stability of the relevant protein complexes. Small molecules derived from BML-210 and the compound NKL54 illustrate this complexity: these agents can influence MEF2-dependent transcription indirectly by altering HDAC-associated regulatory programs rather than acting as validated MEF2-selective inhibitors [56].

### 3.3. The Post-Translational Modification That Affects Transcriptional Activity of MEF2s

There is a complex relationship between the post-translational modification and the stability and transcriptional activity of MEF2. Various modifications and sites can exert either positive or negative regulatory effects on the transcriptional activity of MEF2, which is regulated by a variety of enzymes. The acetylation of MEF2s, regulated by the interactors HDACs and P300/CBP, can affect the activity of MEF2 transcriptionally activated downstream genes [57,58]. For instance, the acetylation of Lys4 plays a significant role in the P300-dependent activation of MEF2C by enhancing the DNA-binding affinity [51]. The modification of key sites of transcription factors may affect the ability of MEF2s to bind to other proteins and thus affect the transcriptional activity of MEF2s. Consequently, mutations occurring in the specific portion of MEF2s can result in positive or negative effects on the transcriptional activities of MEF2s and the outcome is contingent upon the stability of the respective complexes within the epigenetic context and specific cell lineage. The small molecule BML-210, specifically designed to bind MEF2, can inhibit the interaction between MEF2 and class IIa HDACs, which induces the acetylation of MEF2 and histones, leading to growth inhibition, apoptosis and differentiation in leukemia cells [59].

Furthermore, phosphorylation modifications, regulated by various kinases and phosphatases, can also affect the interaction with transcriptional co-regulators and the DNA-binding affinity [58]. Casein kinase II and protein kinase A phosphorylate the MADS domain of MEF2 and influence its DNA-binding affinity. The phosphorylation will further promote class II histone deacetylases (HDACs), dissociating from the MEF2 DNA-binding domain and complexes, which play a role in chromatin stability, transcription initiation, and the rate of transcription [60]. Phosphorylation of MEF2A/D at Ser408/Ser444 by the cyclin-dependent kinase 5 (Cdk5) can subsequently inhibit the Cdk5-dependent apoptosis process [53].

Other post-translational modifications, such as sumoylation, also affect MEF2 transcriptional activity. It should be noted that the effects of different modification sites may interact and be regulated by other transcriptional regulators, especially modifications at adjacent sites that can interact and affect the function of MEF2C. For instance, phosphorylation at S396 of MEF2C promotes SUMOylation at K391, which recruits an unidentified corepressor and inhibits transcription [54]. As an important positive regulator of MEF2-dependent transcription, calcineurin has been shown to phosphorylate MEF2D by controlling SUMOylation of Lys-439; furthermore, in SUMOylation-deficient MEF2D mutant samples, calcineurin has a minimal negative effect on MEF2D transcriptional activity [58]. In addition, the status of the modification site may also be affected by the cellular environment and signaling pathways, thereby further regulating the transcriptional activity of MEF2 [20].

## 4. The MEF2s Associated with Malignant Biological Behavior

The altered expression and activity of MEF2 members are observed in different cancer types, but their functions cannot be classified uniformly as oncogenic or tumor suppressive. Increasing evidence links MEF2 proteins to cancer-related processes such as proliferation, apoptosis, epithelial–mesenchymal transition, angiogenesis, immune escape, and therapy resistance (Table 1). MEF2B is now also included because recurrent MEF2B mutations have important roles in B-cell lymphoma [6,24]. The biological effect of each MEF2 member depends on the regulated target genes, interacting cofactors, signaling context, and tumor lineage.

### 4.1. Proliferation and Apoptosis

**Figure 3:**
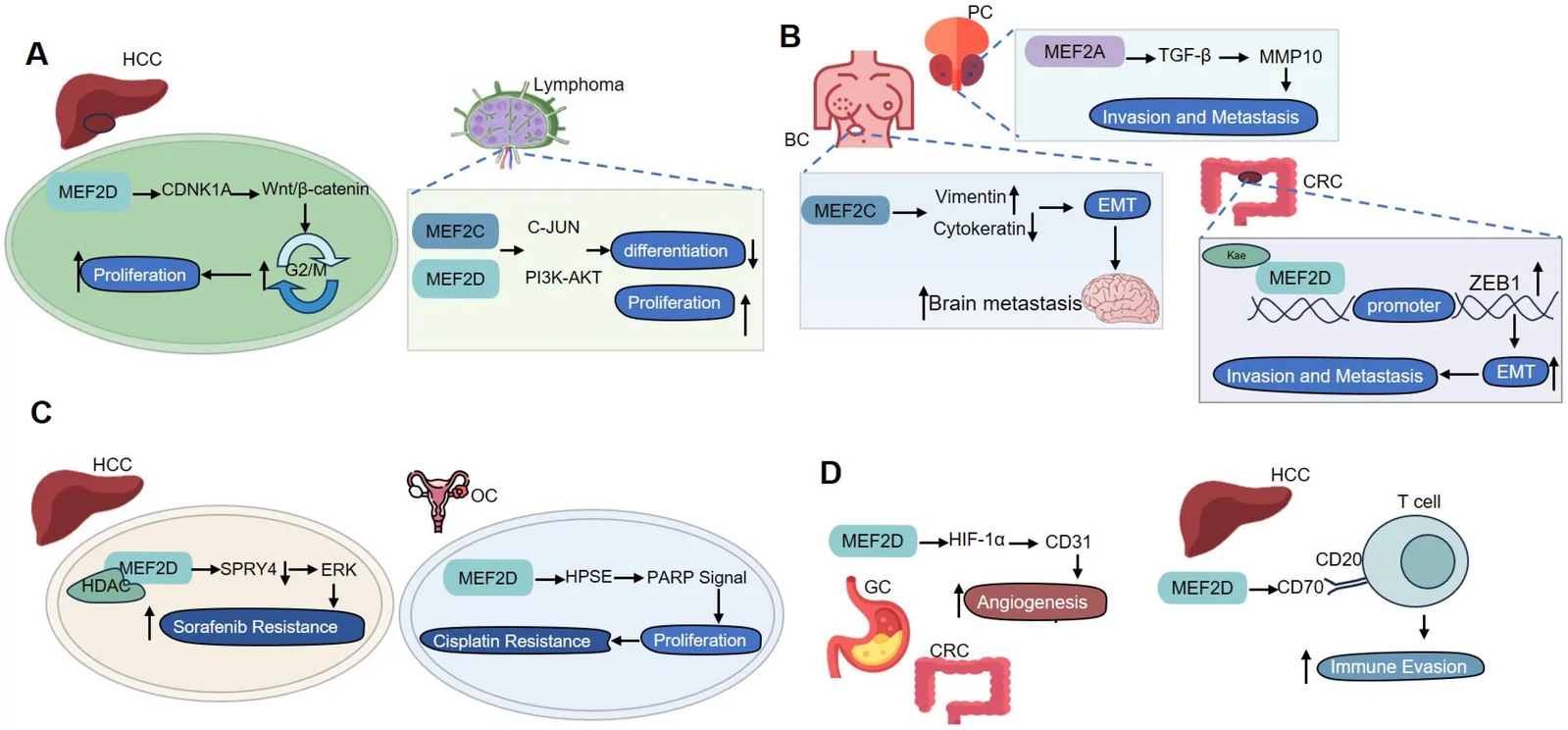
MEF2 family members are involved in various biological functions, including proliferation and differentiation (**A**), invasion and metastasis (**B**), drug resistance (**C**), and angiogenesis and immune escape (**D**). ↑: upregulation; ↓: down-regulation.

In the MEF2 family, MEF2C and MEF2D are the most common factors related to promoting cancer cell proliferation and inhibiting apoptosis. MEF2D mainly promotes cell proliferation in several types of cancers, including lung cancer [74], T-cell lymphoma [75], prostate cancer [76], gastric cancer [77] and HCC cells [70] (Figure 3A). For example, in non-small-cell lung cancer, the upregulation of MEF2D under inflammatory conditions has a positive effect on cancer cell proliferation and migration and may further promote malignant tumor development by affecting the cancer microenvironment and cell biological behavior [74]. In gastric cancer, miR-19 is significantly decreased, which induces a high expression of MEF2D and then activates the classic Wnt/β-catenin pathway and promotes cell proliferation [77]. In PC3 prostate cancer cells, MEF2 increased lncRNA PCGEM1 promoter activity and upregulated PCGEM1 expression. Then, this lncRNA promotes downregulation of tumor suppressor miR-148a, promotes proliferation and reduces apoptosis of PC3 prostate cancer cells [76]. Furthermore, MEF2D overexpression was also found to promote HCC cell proliferation. MEF2D-positive HCC cells exhibited higher proliferation rates compared to MEF2D-negative cells by activating the Wnt/β-catenin signaling pathway, which is positively related to cell proliferation [70].

In non-solid tumors, the role of MEF2D in promoting tumor cell proliferation and inhibiting differentiation has also been observed. In T-cell lymphoma, MEF2D regulates Sh2d1a expression in a DNA binding-dependent manner as a transcriptional repressor, which prevents differentiation of antigen-specific CD4 T cells into germinal center T follicular helper (GC-Tfh) cells and impairs tumor cell growth rate [75]. The c-Jun promoter is recognized by the conserved MEF2 motif in MEF2A and MEF2D and is involved in regulating cell proliferation in several cell types [68,78,79]. In high serum conditions, the DAZAP1/MEF2D fusion protein regulates serum-induced c-Jun expression, whereby MEF2 regulates the cell cycle and leads to a slight reduction in apoptosis in acute lymphoblastic leukemia [68].

The detailed mechanisms by which MEF2C promotes cell proliferation are different in several kinds of cancer. In Rhabdomyosarcoma cells, different alternatively spliced forms of MEF2C exhibit distinct transcriptional activities. The expression of the MEF2Cα1 exon has been shown to inhibit differentiation in the cells. The expression of the MEF2Cα1 exon in RMS cells contributes to the lack of differentiation observed in those cells. On the other hand, the inclusion of the α2 exon enhances MEF2C activity by facilitating the recruitment of various histone deacetylases (HDACs) to target gene promoters. The repair of SRPK3 or MEF2Cα2 in RMS cells can improve differentiation and inhibit proliferation and tumorigenic growth [31]. In CTCL cells, MEF2C is a pro-proliferative factor by targeting MiR-223 to promote cell growth and clonogenic potential [80]. However, some reports indicate that MEF2C inhibits tumor cell proliferation by PKC and p38 MAPK signaling in vitro and in vivo, which is contradictory to previous studies that showed that MEF2C promotes proliferation in HCC [68,71]. Another study has reported that nuclear MEF2C promotes VEGF-mediated invasion and angiogenesis of HCC cells, whereas cytoplasmic MEF2C sequesters β-catenin, reducing the capacity of β-catenin to promote cell proliferation [81,82].

### 4.2. Migration and Metastasis

In the complex progression of tumors, three members of the MEF2 family, MEF2A, MEF2C and MEF2D significantly promote tumor EMT, migration and metastasis by regulating the expression of genes related to cell movement and morphological changes [64]. In HCC, TGF-β1 activates Akt, which phosphorylates MEF2 and enhances its nuclear translocation and transcriptional activity. This pathway regulates the expression and activity of MEF2A, MEF2C and MEF2D and then impacts HCC cell proliferation, invasion, and metastasis. Thus, targeting the TGF-β1/Akt/MEF2 signaling pathway holds potential for HCC treatment and offers new therapeutic avenues [83].

MEF2A is involved in fibroblast growth factor signal transduction in prostate and breast cancer epithelial cells [84,85,86,87]. In terms of mechanism, TGF-β is upstream of MEF2A and activates MEF2A transcriptional activity by directly catalyzing the acetylation of MEF2A and indirectly inhibiting the HDAC activity [84,85]. Highly transcriptionally active MEF2A is a direct activator of MMP10 transcription, which degrades the extracellular matrix, thereby promoting malignant tumor invasion and metastasis of prostate and breast cancer [85,88].

MEF2C is mainly expressed in malignant duct cells and other scattered malignant epithelial cells in primary breast cancer. These cells have a low expression of epithelial and tumor marker pan-cytokeratin, which means that MEF2C-expressing cells have lost their epithelial characteristics [64,89,90]. In-depth research found that MEF2C plays a role not only in the first step of metastasis—escaping from the primary site—but also in the step of colonization, where the progress is accompanied by losing, restoring and continuing expression of epithelial characteristics [64]. In addition, MEF2C was found to promote breast cancer metastasis through the blood–brain barrier by affecting the expression of downstream miR-802-5p and miR-194-5p in the blood [91].

In CRC, MEF2D was found to be related to tumor metastasis and involved in regulating the EMT phenotype, in which EGF and hypoxia-induced EMT behaviors are dependent on MEF2D for function [92]. Mechanistically, MEF2D acts as a central integrator of microenvironmental signals to activate ZEB1, through which MEF2D regulates cancer cell EMT. The therapeutic target of MEF2D can be further studied in the future [92] (Figure 3B).

### 4.3. Drug Resistance

MEF2C and MEF2D are both related to treatment resistance in HCC [93], OC [69], ALL [65] and AML [94], among which the mechanism of drug resistance in leukemia has been studied in depth. In the study of steroid treatment for patients with Early T-cell precursor acute lymphoblastic leukemia (ETP-ALL), MEF2C expression was significantly higher in steroid-resistant LOUCY cells compared to steroid-sensitive cells. MEF2C antagonizes Notch signaling indirectly and activates mutations in IL-7R signaling pathway components and BCL2, which induce steroid resistance more frequently [65]. The SIK inhibition blocks MEF2C function by reducing phospho-MEF2C protein levels and decreasing expression of MEF2C target genes, which synergistically reversed the MEF2C-induced steroid resistance of LOUCY cells [65].

In addition, MEF2C is a highly expressed and active TF gene in HCC models [14,95]. Mitogen-inducible gene 6 (*MIG6*) is upregulated in gefitinib-resistant cancer tissues and cell lines, which can promote the growth of HCC cells by increasing the activity of Caspase-MEF2C, which is the upstream transcriptional factor of MIG6. MEF2C knockdown inhibited the gefitinib resistance and limited the proliferation of HCC by blocking the MIG6/Caspase-3 signal in both in vitro and in vivo experiments [93].

MEF2D correlates with sorafenib resistance in HCC and high expression of MEF2D correlates with poor prognosis of HCC patients treated with sorafenib. In vivo experiments show that overexpression of MEF2D reduces sorafenib-treatment sensitivity, while depletion of MEF2D decreases tumor cell survival and tumor growth rates under the treatment of sorafenib [96]. Further in vitro mechanism experiments exhibit that MEF2D forms a complex with the co-repressor HDAC4, which represses SPRY4 expression and activates ERK and subsequently promotes sorafenib resistance in HCC cells. Inhibiting HDAC4 reverses MEF2D-mediated SPRY4 suppression, sensitizes HCC cells to sorafenib-induced apoptosis and inhibits liver tumor growth in mice treated with sorafenib [96].

In ovarian carcinoma (OC), MEF2D serves as an oncogenic driver associated with cisplatin resistance. In a retrospective of patients with OC, a high level of MEF2D was strongly associated with resistance and poor prognosis [69]. Further mechanism research shows that MEF2D can directly bind to the promoter regions of DNA repair genes, anti-apoptotic genes and drug resistance genes, thereby promoting the development of cisplatin resistance because DNA damage caused by chemotherapy drugs is not effectively repaired [69] (Figure 3C). In addition to solid tumors, children with relapsed or primary refractory B-cell acute lymphoblastic leukemia (B-ALL) have also been found to have an MEF2D-BCL9 translocation, and they are resistant to dexamethasone treatment for this specific subtype of leukemia [97].

### 4.4. Angiogenesis and Immune Evasion

MEF2A, MEF2C, and MEF2D are also expressed in endothelial cells and are key mediators involved in sprouting angiogenesis and remodeling the vascular downstream of VEGFA [88,98]. They promote the expression of Dll4 and some other key genes involved in sprouting angiogenesis in tumor vasculogenesis by specifically enriching for the genes related to sprouting angiogenesis via the dissociation of MEF2 and HDAC, and the combination of EP300 and MEF2 [88]. High levels of Dll4 expression are required for the formation of leading tip endothelial cells, which in turn triggers Notch signaling and promotes vascular [88].

The dual role of MEF2C in tumors has also been reported in HCC because of the subcellular distribution [71,99]. The cytoplasmic MEF2C dissociates with β-catenin in the cytoplasm and reduces the ability of β-catenin to promote cell proliferation, while the nuclear MEF2C promotes invasion and angiogenesis. The nucleus MEF2C plays a role in VEGF-induced vasculogenic mimicry and vasculogenesis through regulation of p38 MAPK and PKC signaling pathways, and further promotes upregulation of the angiogenesis-related biomarker CD31 [71].

MEF2D expression is positively correlated with CD31-positive microvessel density in CRC tissues. MEF2D induces the expression of pro-angiogenic cytokines, which are also downstream mediators of hypoxia-inducible factor (HIF)-1α that drive tumor angiogenesis in CRC. These findings suggest that targeting the HIF-1α/MEF2D axis may be a potential therapeutic strategy for the treatment of CRC [100,101].

MEF2D can also regulate immune evasion in cancer treatment. It increases T-cell activation and inhibits T regulatory (Treg) cell suppressive function in HCC [102]. In murine HCC tumors, p300-induced MEF2D acetylation blocked T-cell-mediated antitumor immunity by regulating the expression of CD70 and activating the CD70-CD27 signaling (Figure 3D).

## 5. Correlation of Clinicopathological Characteristics and Therapeutic Potential

The combination of transcription factors and a specific binding site depends on the thermodynamic stability of the TF–DNA complex, multidimensional DNA/chromatin architecture, the cooperative action of other TFs and co-activators at nearby or overlapping sites. In order to target the TF signal, intervention methods can be designed from the following perspectives, including small molecule inhibitor (SMI) targeting activity and antibody therapies blocking the molecular and gene therapies by regulating gene expression. For example, OTX-**2002**—an mRNA therapeutic delivered via lipid nanoparticles (LNPs) and designed to downregulate transcription factor MYC expression—showed antitumor activity in patients with hepatocellular carcinoma and other solid tumor types in some clinical trials [103,104].

**Figure 4:**
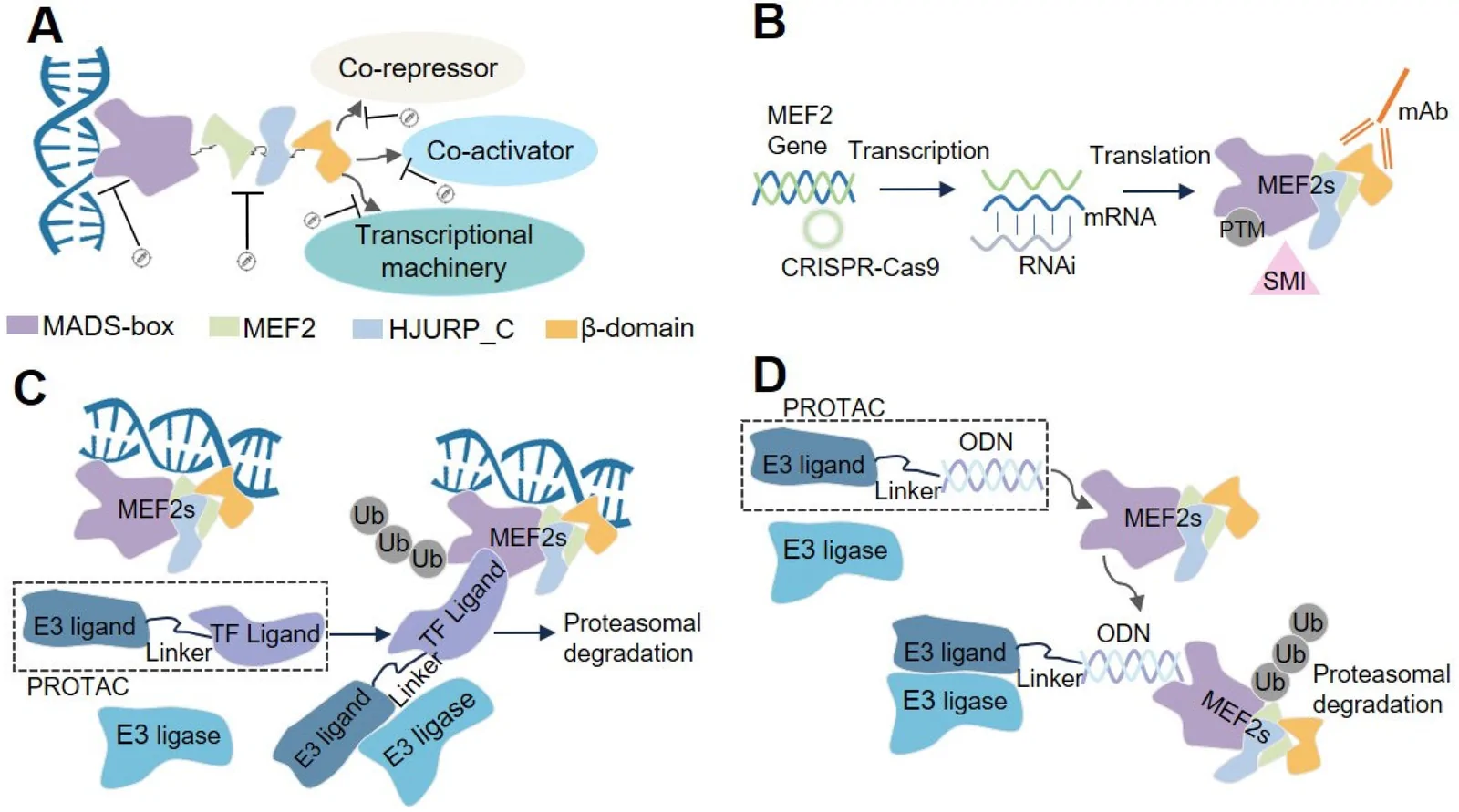
The potential strategies to modulate MEF2-dependent transcription. (**A**) MEF2 family member regulates target genes by forming complexes with DNA and transcriptional co-regulators; these complexes may be modulated directly or indirectly. (**B**) There are four main approaches to suppress MEF2s: by CRISPR-Cas9 genetic editing; by RNAi genetic manipulation; by conventional SMIs protein inhibiting activity; or by monoclonal antibodies neutralization. (**C**) PROTACs are bifunctional molecules interacting with the protein of interest while engaging an E3 ubiquitin ligase to degrade the target protein selectively. This concept was established for other targets and remains hypothetical for MEF2 until high-affinity MEF2 ligands are developed. (**D**) Model of DNA oligonucleotide-base (ODN) TF-PROTACs are designed to target MEF2, where an ODN is linked to an E3 ligand via a linker. The TF-PROTACs of MEF2-specific efficacy, selectivity among MEF2 isoforms, delivery, and safety have not yet been demonstrated.

Based on the molecular structural characteristics of the MEF2 transcription factor family members, we believe that there are several emerging potential strategies to intervene in MEF2 targets to treat tumors. In theory, intervention can be conducted by targeting the binding of transcription factors to chromatin sequences, either directly affecting the level and activity of transcription factors or indirectly regulating transcriptional co-regulatory molecules (Figure 4A). To suppress MEF2s directly, the main ways include CRISPR-Cas9 genetic editing of MEF2, RNAi genetic manipulation, conventional SMIs inhibiting protein activity, and monoclonal antibodies neutralizing protein (Figure 4B). However, the conserved MADS/MEF2 DNA-binding domains make isoform-selective targeting difficult (Figure 1A-C), and systemic suppression could affect normal cardiac, skeletal muscle, neuronal, endothelial, and immune functions. To design a specific target for different MEF2 members, the HJURP_C domain can be the key region that researchers focus on. Therefore, future therapeutic development must address whether a strategy suppresses one MEF2 family member, multiple MEF2 members, or a broader MEF2-associated transcriptional complex. When targeting MEF2 family members, drug toxicity and side effects are issues that require further validation in the future.

PROTACs were originally developed as chimeric molecules that recruit a target protein to an E3 ubiquitin ligase for ubiquitination and degradation [105], and the field has since expanded to many protein classes [106]. In principle, an MEF2-directed degrader would require a ligand that binds the intended MEF2 protein with sufficient affinity and selectivity, an E3 ligase ligand, and a linker that supports ternary-complex formation (Figure 4C). At present, however, no validated MEF2-specific PROTAC has been reported. The PROTACs operate via an event-driven mechanism, catalytically degrading target proteins through proximity-induced ubiquitination without needing active site occupancy—allowing the targeting of undruggable proteins. Targeted therapy against MEF2 protein molecules using the PROTAC system represents a promising direction for future research. Moreover, existing drug-resistance test results indicate that combining MEF2-targeted therapy with conventional chemotherapy may be a key direction for future combination therapy in drug-resistant patients.

Since oligonucleotides are natural ligands of transcription factors, DNA-oligonucleotide-based (ODN) TF-PROTACs can be modified to target different transcription factors by swapping out the DNA part of the chimeric oligo, offering a promising approach for targeting traditionally undruggable transcription factors (Figure 4D). To design PROTACs targeting MEF2, the ODN targeting MEF2 is linked to an E3 ligand via a linker, and TF-PROTACs induce the formation of an E3 ligand-TF-PROTAC-TOI ternary complex, leading to degradation of TFs by the ubiquitin–proteasome system (Figure 4D). Oligonucleotide-based degrader strategies provide another conceptual route for DNA-binding transcription factors. TF-PROTACs and oligo-PROTACs have degraded selected transcription factors such as NF-kappaB family proteins, LEF1, and ERG in experimental systems [107,108] and an oligo-PROTAC strategy has also been reported for activated STAT3 [109]. These platforms suggest a possible path for MEF2, but MEF2-specific ODN degraders must still overcome sequence selectivity, nuclear delivery, degradation efficiency, and off-target transcriptional effects (Figure 4D).

It is worth noting that there have been many studies on the relationship between MEF2 and the clinical prognosis of tumor patients and its predictive value (Table 2). Existing data suggest that MEF2A, MEF2B, MEF2C, and MEF2D can have prognostic or clinicopathological value in selected cancers. And a research analysis showed that increased MEF2A mRNA expression is linked with the poor prognosis of patients with gastric cancer [103]. The aberrant high expression of MEF2A in human CRC tissues is correlated with unfavorable prognosis and metastasis, by the mechanism of inducing EMT-related TFs and Wnt/β-catenin signaling [104]. MEF2D mRNA expression is correlated with poorer survival of patients with pancreatic cancer [105]. Gastric cancer patients with upregulated MEF2D expression had aggressive clinical features and a poor prognosis, including OS and PFS [106].

## 6. Conclusions

The MEF2 families are a group of highly conserved molecules that play pivotal roles in the regulation of gene expression. This review aims to provide an overview of MEF2 family members, various regulatory mechanisms, involvement in tumor-related processes and clinical application. Specifically, the MEF2 family consists of four members: *MEF2A*, *MEF2B*, *MEF2C* and *MEF2D*, which show relatively conserved gene sequence and domains. These transcription factors share similar domains, including the MADS-box and MEF2 domains that serve as DNA- or protein-binding domains, as well as a less conserved C-terminal domain, which is responsible for binding to other protein molecules [10]. By its distinctive DNA-binding capacity, MEF2 interacts with specific binding sites in the transcription start sites of target genes, then plays a role as a transcription activator or repressor depending on the tumor microenvironment [38].

MEF2 proteins exhibit dual functionality as transcriptional activators or repressors depending on MEF2 protein stability, DNA binding affinity, and transcriptional activity modulated by their interactions with co-regulatory proteins and post-translational modification, including phosphorylation, acetylation, and sumoylation [17]. The co-factors such as HDACs, HATs, P300/CBP and SRF are the common interactors binding to MEF2 and participating in gene regulation as complex protein complexes [33,46,49,50]. This sophisticated molecular machinery involving MEF2 and its co-factors governs the physiological and pathological processes of gene regulation, cellular function and tumor progression. This summary highlights the significant contributions of MEF2 factors to cancer biology and prognosis value, which provides valuable insights and potential therapeutic strategies for targeting MEF2-mediated pathways.

MEF2A, MAF2B, MEF2C, and MEF2D are dysregulated and participate in malignant progression in various types of cancer. MEF2B is a potent immunohistochemical marker for neoplastic LP cells in nodular lymphocyte-predominant Hodgkin lymphoma [114]. Abnormal expression and activation of MEF2A, MEF2C, and MEF2D can have pivotal effects on tumor initiation, growth, invasion, and metastasis [76]. MEF2 transcription factors can regulate the expression of genes involved in cell-cycle progression, apoptosis, and DNA damage response [64,65,66,67,68]. Dysregulated MEF2 activity, and therefore altered MEF2 activity, can contribute to uncontrolled cell growth and clonal proliferation [64,65,66,67,68]. Moreover, MEF2 factors have been implicated in promoting EMT, which allows cancer cells to acquire invasive and migratory properties, facilitating their spread to distant tissues by menstrual blood [76,79,82]. The MEF2 protein also functions by interacting with other transcriptional regulators and signaling pathways involved in cancer progression; they can interact with oncogenic factors or modulate key signaling pathways, such as Norch, Wnt/β-catenin and PI3K/AKT, to promote tumor growth [66]. However, a few studies suggest that MEF2 factors may also act as tumor suppressors in certain contexts, such as MEF2A, which exhibits tumor suppressive roles by inducing the expression of CDKN1A via KLF4 [108].

Most current studies indicate that MEF2A, MEF2C, and MEF2D are associated with poor prognosis in several cancers and often function in oncogenic programs [18,109]. Current preclinical studies suggest that targeting the MEF2 signaling pathway is a highly attractive strategy. Some model studies provide the clearest support: MEF2C knockdown reduced gefitinib resistance and tumor growth in HCC models [88]. MEF2D depletion sensitized HCC cells to sorafenib and reduced tumor growth in mice [94], and MEF2D overexpression promoted oncogenic malignancy and cisplatin resistance in ovarian carcinoma [89]. The normal cells would be spared or that systemic MEF2 inhibition would be safe; therefore, off-target effects still need to be considered in subsequent treatments.

In the review, we highlight the activity of each MEF2 protein and the different results of MEF2 biofunction, illustrating the pleiotropic effector transcription factors in tumor progression. MEF2-directed treatment should currently be viewed as an investigational strategy. The literature suggests that the transcription factor MEF2 has considerable potential for future tumor treatment, referring to other TF-related treatment strategies. Nevertheless, MEF2-directed treatment should currently be viewed as an investigational strategy. ODN-based TF-PROTACs, O’PROTACs, small molecules such as NKL54, and RNA-based approaches offer useful conceptual frameworks, but MEF2-specific selectivity, delivery, efficacy, and safety must be established before clinical translation [56,107,108,109]. DNA-oligonucleotide-based (ODN) TF-PROTACs targeting MEF2 will be the future direction because of the lower possibility of off-target effects.

In conclusion, the dysregulation of MEF2 transcription factors exhibits a significant role in tumor initiation and progression. The MEF2A, MEF2C and MEF2D participate in cell proliferation, stemness, apoptosis, EMT, and are associated with other malignant signaling pathways, highlighting their potential as cancer therapeutic targets. They also show potential prognostic value in future clinical applications. Further investigations into the molecular mechanisms underlying MEF2-mediated tumorigenesis will provide valuable insights for developing targeted therapies against various types of cancer.

## 7. Future Directions

Future studies should figure out which tumor types are most dependent on a specific MEF2 member, whether MEF2 alteration is a driver or a passenger event, and which normal tissues are vulnerable to MEF2 inhibition. Priority areas include isoform-selective perturbation, tumor-selective delivery of RNA or oligonucleotide degraders, validation of small molecules that alter MEF2-dependent transcription, and preclinical testing in models that capture tumor lineage and immune context.

## Figures and Tables

**Table 1:** Representative cellular functions and mechanisms of MEF2 proteins in cancers.

Molecular	Role of MEF2 Protein	Cancer Type	Alteration of MEF2	Effects of MEF2 Alteration
MEF2A [61]	Tumor suppressor	RCC	Modulation of Wnt/β-catenin signaling.	MEF2A overexpression inhibits RCC proliferation, invasion, migration and epithelial–mesenchymal transition (EMT)
MEF2A [22]	Oncogene	GC	Activated p38MAPK leads to increased MEF2a-dependent expression of GLUT-4	Promotes glycolysis and proliferation of gastric tumor cells.
MEF2A [55]	Oncogene	CRC	MEF2A promotes ZEB2, CTNNB1, EMT-related TFs and Wnt/β-catenin signaling.	Increases the frequency of tumor formation and metastasis.
MEF2B [62]	Oncogene	DLBCL	Mutations of MEF2B contribute to lymphomagenesis by deregulating BCL6 expression.	MEF2B increases cell proliferation by promoting cell-cycle progression.
MEF2C [63]	Oncogene	Immature T-ALL	Increased expression because of rearrangements or alterations affecting interacting proteins.	Inhibition of differentiation.
MEF2C [64]	Oncogene	BC	MEF2C expression in malignant cells extravasating into the brain and still close to blood–brain barrier (BBB) microvessels.	Promotes breast cancer brain metastases.
MEF2C [65]	Oncogene	ALL	MEF2C activates a B cell transcriptional program including RUNX1, GATA3, LMO2, BCL2 and the IL-7R to boost cell survival.	Blocks early T cell differentiation and promote mixed phenotype acute leukemia.
MEF2C [66]	Oncogene	PC	Low-expression YY1 increases MEF2C expression and downregulates MMP10.	Promotes invasiveness.
MEF2D [67]	Oncogene	AML	MEF2D represses CEBPE-centered myeloid differentiation program by binding to and suppresses the chromatin accessibility of CEBPE cis-regulatory regions.	Promotes cell stemness.
MEF2D [68]	Oncogene	B-ALL	Fusion with DAZAP1 protein.	Promotes proliferation and inhibits apoptosis.
MEF2C, MEF2D [36,47,69,70,71,72]	Oncogene	HCC	MEF2D transactivates Itgb1 (coding β1-integrin) and Itgb4 (coding β4-integrin) to provide superior anti-metastatic efficacy.	Promotes pro-metastatic signaling.
MEF2C, MEF2D [13]	Tumor suppressor	LMS	The increased HDAC4 and PI3K/AKT activity decreased MEF2 activity and abundance.	Promotes cell proliferation and anchorage-independent growth.
MEF2C, MEF2D [31,73]	Tumor suppressor	RMS	MEF2 target genes such as PTEN is repressed in soft tissue sarcomas.	Promotes differentiation and inhibits cell proliferation and cell migration.

CRC: Colorectal cancer; DLBCL: diffuse large B-cell lymphoma; GC: Gastric cancer; PC: pancreatic cancer; HCC: Hepatocellular carcinoma; T-ALL: T cell acute lymphoblastic leukemia; AML: Acute myeloid leukemia; T-cell ALL: T-acute lymphoblastic leukemia; BC: Breast cancer; B-ALL: B-cell acute lymphoblastic leukemia; LMS: Lipo- and leiomyosarcoma; RMS: Rhabdomyosarcoma.

**Table 2:** The clinical evidence linking MEF2 dysregulation with clinicopathological features and prognosis.

Cancer	MEF2	Role in Cancer	Expression Status	Relationship with Patient Survival and Prognosis	Relevant Clinical Factors
AML	MEF2C [15]	Promotive	High mRNA expression	High level of MEF2C is an independent prognostic factor for worse OS	Therapy resistance
BCL	MEF2B [5]	Promotive	Mutant MEF2BD83V	MEF2BD83V markedly related to the less incidence of lymphomas	NA
B-ALL	MEF2D [12]	Promotive	MEF2D fusion	High level of MEF2D is an independent prognostic factor for worse OS and EFS	NA
CRC	MEF2A [55]	Promotive	High mRNA and protein expression	High level of MEF2A is an independent prognostic factor for worse OS	Poor histological differentiation, late TNM stage, lymph node metastasis, distant metastasis,
CRC	MEF2D [92]	Promotive	High mRNA and protein expression	High level of MEF2D is an independent prognostic factor for worse OS	Lymph node metastasis, distant metastasis and recurrence.
GC	MEF2D [110]	Promotive	High mRNA and protein expression	High level of MEF2D is an independent prognostic factor for worse OS and PFS	Distant liver metastasis
GC	MEF2C [111]	Promotive	High mRNA expression	High level of MEF2C is an independent prognostic factor for worse OS	Negatively correlated with tumor mutation burden (TMB) and microsatellite instability (MSI)
OC	MEF2D [69]	Promotive	High mRNA and protein expression	High level of MEF2D is an independent prognostic factor for worse OS	Clinical stage, pathological grade, histologic type and cisplatin-resistance.
PC	MEF2D [16,112]	Promotive	High mRNA expression	High level of MEF2D is an independent prognostic factor for worse OS	NA
RCC	MEF2A [61]	NA	Low mRNA and protein expression	Low level of MEF2A is an independent prognostic factor for worse OS	NA
T-cell ALL	MEF2C [113]	Promotive	High mRNA expression	High level of MEF2C is an independent prognostic factor for worse OS and EFS	Immature immunophenotype.

CRC: Colorectal cancer; EC: Esophageal carcinoma; GC: Gastric cancer; PC: pancreatic cancer; AML: Acute myeloid leukemia; T-cell ALL: T-acute lymphoblastic leukemia; BCL: B-cell lymphomas; OS: Overall survival; EFS: Event-free survival; NA indicates that direct evidence is not available.

## Data Availability

No new data were created or analyzed in this study. Data sharing is not applicable to this article.

## References

[1] Gossett L.A., Kelvin D.J., Sternberg E.A., Olson E.N. (1989). A new myocyte-specific enhancer-binding factor that recognizes a conserved element associated with multiple muscle-specific genes. Mol. Cell. Biol..

[2] Lisek M., Przybyszewski O., Zylinska L., Guo F., Boczek T. (2023). The Role of MEF2 Transcription Factor Family in Neuronal Survival and Degeneration. Int. J. Mol. Sci..

[3] Pon J.R., Marra M.A. (2016). MEF2 transcription factors: Developmental regulators and emerging cancer genes. Oncotarget.

[4] Wales S., Hashemi S., Blais A., McDermott J.C. (2014). Global MEF2 target gene analysis in cardiac and skeletal muscle reveals novel regulation of DUSP6 by p38MAPK-MEF2 signaling. Nucleic Acids Res..

[5] Brescia P., Schneider C., Holmes A.B., Shen Q., Hussein S., Pasqualucci L., Basso K., Dalla-Favera R. (2018). MEF2B Instructs Germinal Center Development and Acts as an Oncogene in B Cell Lymphomagenesis. Cancer Cell.

[6] Materna S.C., Sinha T., Barnes R.M., Lammerts van Bueren K., Black B.L. (2019). Cardiovascular development and survival require Mef2c function in the myocardial but not the endothelial lineage. Dev. Biol..

[7] Wang Y., Wang Y. (2025). Transcription Factor Dynamics in Neuroendocrine Prostate Cancer Development. Holist. Integr. Oncol..

[8] Hobson G.M., Krahe R., Garcia E., Siciliano M.J., Funanage V.L. (1995). Regional chromosomal assignments for four members of the MADS domain transcription enhancer factor 2 (MEF2) gene family to human chromosomes 15q26, 19p12, 5q14, and 1q12-q23. Genomics.

[9] Wu W., de Folter S., Shen X., Zhang W., Tao S. (2011). Vertebrate paralogous MEF2 genes: Origin, conservation, and evolution. PLoS ONE.

[10] Chen Z., Wang Q., Zhang H., Ma X., Wu W., Cheng N., Zhang J., Zhou A., Li Y., Meng G. (2021). Purification, crystallization, and X-ray diffraction analysis of myocyte enhancer factor 2D and DNA complex. Protein Expr. Purif..

[11] Potthoff M.J., Olson E.N. (2007). MEF2: A central regulator of diverse developmental programs. Development.

[12] Ohki K., Kiyokawa N., Saito Y., Hirabayashi S., Nakabayashi K., Ichikawa H., Momozawa Y., Okamura K., Yoshimi A., Ogata-Kawata H. (2019). Clinical and molecular characteristics of MEF2D fusion-positive B-cell precursor acute lymphoblastic leukemia in childhood, including a novel translocation resulting in MEF2D-HNRNPH1 gene fusion. Haematologica.

[13] Di Giorgio E., Clocchiatti A., Piccinin S., Sgorbissa A., Viviani G., Peruzzo P., Romeo S., Rossi S., Dei Tos A.P., Maestro R. (2013). MEF2 is a converging hub for histone deacetylase 4 and phosphatidylinositol 3-kinase/Akt-induced transformation. Mol. Cell. Biol..

[14] Zhu G.Q., Tang Z., Huang R., Qu W.F., Fang Y., Yang R., Tao C.Y., Gao J., Wu X.L., Sun H.X. (2023). CD36(+) cancer-associated fibroblasts provide immunosuppressive microenvironment for hepatocellular carcinoma via secretion of macrophage migration inhibitory factor. Cell Discov..

[15] Menshawy N.E., El-Ghonemy M.S., Ebrahim M.A., Fahmi M.W., Saif M., Denewer M., El-Ashwah S. (2024). Aberrant ecotropic viral integration site-1 (EVI-1) and myocyte enhancer factor 2 C gene (MEF2C) in adult acute myeloid leukemia are associated with adverse t (9:22) & 11q23 rearrangements. Ann. Hematol..

[16] Homminga I., Pieters R., Langerak A.W., de Rooi J.J., Stubbs A., Verstegen M., Vuerhard M., Buijs-Gladdines J., Kooi C., Klous P. (2011). Integrated transcript and genome analyses reveal NKX2-1 and MEF2C as potential oncogenes in T cell acute lymphoblastic leukemia. Cancer Cell.

[17] Wu Y., Dey R., Han A., Jayathilaka N., Philips M., Ye J., Chen L. (2010). Structure of the MADS-box/MEF2 domain of MEF2A bound to DNA and its implication for myocardin recruitment. J. Mol. Biol..

[18] Estrella N.L., Desjardins C.A., Nocco S.E., Clark A.L., Maksimenko Y., Naya F.J. (2015). MEF2 transcription factors regulate distinct gene programs in mammalian skeletal muscle differentiation. J. Biol. Chem..

[19] Ewen E.P., Snyder C.M., Wilson M., Desjardins D., Naya F.J. (2011). The Mef2A transcription factor coordinately regulates a costamere gene program in cardiac muscle. J. Biol. Chem..

[20] Liu B., Ou W.C., Fang L., Tian C.W., Xiong Y. (2023). Myocyte Enhancer Factor 2A Plays a Central Role in the Regulatory Networks of Cellular Physiopathology. Aging Dis..

[21] Lu H., Liu B., Zhang F.J., Zhang J., Dong R., Chen L., Qu D.M., Lu Y., Yu B.W. (2014). The E3 ligase APC/C-Cdh1 regulates MEF2A-dependent transcription by targeting SUMO-specific protease 2 for ubiquitination and degradation. Cell Cycle.

[22] Liu J., Wen D., Fang X., Wang X., Liu T., Zhu J. (2015). p38MAPK Signaling Enhances Glycolysis Through the Up-Regulation of the Glucose Transporter GLUT-4 in Gastric Cancer Cells. Cell Physiol. Biochem..

[23] Barbosa A.C., Kim M.S., Ertunc M., Adachi M., Nelson E.D., McAnally J., Richardson J.A., Kavalali E.T., Monteggia L.M., Bassel-Duby R. (2008). MEF2C, a transcription factor that facilitates learning and memory by negative regulation of synapse numbers and function. Proc. Natl. Acad. Sci. USA.

[24] Wilker P.R., Kohyama M., Sandau M.M., Albring J.C., Nakagawa O., Schwarz J.J., Murphy K.M. (2008). Transcription factor Mef2c is required for B cell proliferation and survival after antigen receptor stimulation. Nat. Immunol..

[25] Wang L., Huang P., Near D., Ravi K., Xu Y., Liu J., Qian L. (2020). Isoform Specific Effects of Mef2C during Direct Cardiac Reprogramming. Cells.

[26] Le Meur N., Holder-Espinasse M., Jaillard S., Goldenberg A., Joriot S., Amati-Bonneau P., Guichet A., Barth M., Charollais A., Journel H. (2010). MEF2C haploinsufficiency caused by either microdeletion of the 5q14.3 region or mutation is responsible for severe mental retardation with stereotypic movements, epilepsy and/or cerebral malformations. J. Med. Genet..

[27] Borlot F., Whitney R., Cohn R.D., Weiss S.K. (2019). MEF2C-related epilepsy: Delineating the phenotypic spectrum from a novel mutation and literature review. Seizure.

[28] Piasecka A., Sekrecki M., Szczesniak M.W., Sobczak K. (2021). MEF2C shapes the microtranscriptome during differentiation of skeletal muscles. Sci. Rep..

[29] Dodou E., Xu S.M., Black B.L. (2003). mef2c is activated directly by myogenic basic helix-loop-helix proteins during skeletal muscle development in vivo. Mech. Dev..

[30] Rashid A.J., Cole C.J., Josselyn S.A. (2014). Emerging roles for MEF2 transcription factors in memory. Genes. Brain Behav..

[31] Zhang M., Zhu B., Davie J. (2015). Alternative splicing of MEF2C pre-mRNA controls its activity in normal myogenesis and promotes tumorigenicity in rhabdomyosarcoma cells. J. Biol. Chem..

[32] Zhao X., Sternsdorf T., Bolger T.A., Evans R.M., Yao T.P. (2005). Regulation of MEF2 by histone deacetylase 4- and SIRT1 deacetylase-mediated lysine modifications. Mol. Cell. Biol..

[33] Gregoire S., Xiao L., Nie J., Zhang X., Xu M., Li J., Wong J., Seto E., Yang X.J. (2007). Histone deacetylase 3 interacts with and deacetylates myocyte enhancer factor 2. Mol. Cell. Biol..

[34] Okamoto S., Krainc D., Sherman K., Lipton S.A. (2000). Antiapoptotic role of the p38 mitogen-activated protein kinase-myocyte enhancer factor 2 transcription factor pathway during neuronal differentiation. Proc. Natl. Acad. Sci. USA.

[35] Vrailas-Mortimer A., del Rivero T., Mukherjee S., Nag S., Gaitanidis A., Kadas D., Consoulas C., Duttaroy A., Sanyal S. (2011). A muscle-specific p38 MAPK/Mef2/MnSOD pathway regulates stress, motor function, and life span in Drosophila. Dev. Cell.

[36] Xiang J., Zhang N., Du A., Li J., Luo M., Wang Y., Liu M., Yang L., Li X., Wang L. (2023). A Ubiquitin-Dependent Switch on MEF2D Senses Pro-Metastatic Niche Signals to Facilitate Intrahepatic Metastasis of Liver Cancer. Adv. Sci. (Weinh.).

[37] Gonczi M., Teixeira J.M.C., Barrera-Vilarmau S., Mediani L., Antoniani F., Nagy T.M., Feher K., Raduly Z., Ambrus V., Tozser J. (2023). Alternatively spliced exon regulates context-dependent MEF2D higher-order assembly during myogenesis. Nat. Commun..

[38] Dantas Machado A.C., Cooper B.H., Lei X., Di Felice R., Chen L., Rohs R. (2020). Landscape of DNA binding signatures of myocyte enhancer factor-2B reveals a unique interplay of base and shape readout. Nucleic Acids Res..

[39] Andres V., Cervera M., Mahdavi V. (1995). Determination of the consensus binding site for MEF2 expressed in muscle and brain reveals tissue-specific sequence constraints. J. Biol. Chem..

[40] Slepak T.I., Webster K.A., Zang J., Prentice H., O’Dowd A., Hicks M.N., Bishopric N.H. (2001). Control of cardiac-specific transcription by p300 through myocyte enhancer factor-2D. J. Biol. Chem..

[41] Taylor M.V., Hughes S.M. (2017). Mef2 and the skeletal muscle differentiation program. Semin. Cell Dev. Biol..

[42] Wei J., Joshi S., Speransky S., Crowley C., Jayathilaka N., Lei X., Wu Y., Gai D., Jain S., Hoosien M. (2017). Reversal of pathological cardiac hypertrophy via the MEF2-coregulator interface. JCI Insight.

[43] Zhang C.L., McKinsey T.A., Olson E.N. (2001). The transcriptional corepressor MITR is a signal-responsive inhibitor of myogenesis. Proc. Natl. Acad. Sci. USA.

[44] Chaudhary R., Agarwal V., Kaushik A.S., Rehman M. (2021). Involvement of myocyte enhancer factor 2c in the pathogenesis of autism spectrum disorder. Heliyon.

[45] Xu J., Li C., Kang X. (2023). The epigenetic regulatory effect of histone acetylation and deacetylation on skeletal muscle metabolism-a review. Front. Physiol..

[46] Ichihara M., Kamiya T., Hara H., Adachi T. (2018). The MEF2A and MEF2D function as scaffold proteins that interact with HDAC1 or p300 in SOD3 expression in THP-1 cells. Free Radic. Res..

[47] McKinsey T.A., Zhang C.L., Olson E.N. (2002). MEF2: A calcium-dependent regulator of cell division, differentiation and death. Trends Biochem. Sci..

[48] Lahm A., Paolini C., Pallaoro M., Nardi M.C., Jones P., Neddermann P., Sambucini S., Bottomley M.J., Lo Surdo P., Carfi A. (2007). Unraveling the hidden catalytic activity of vertebrate class IIa histone deacetylases. Proc. Natl. Acad. Sci. USA.

[49] Di Giorgio E., Wang L., Xiong Y., Akimova T., Christensen L.M., Han R., Samanta A., Trevisanut M., Bhatti T.R., Beier U.H. (2020). MEF2D sustains activation of effector Foxp3+ Tregs during transplant survival and anticancer immunity. J. Clin. Investig..

[50] He J., Ye J., Cai Y., Riquelme C., Liu J.O., Liu X., Han A., Chen L. (2011). Structure of p300 bound to MEF2 on DNA reveals a mechanism of enhanceosome assembly. Nucleic Acids Res..

[51] Angelelli C., Magli A., Ferrari D., Ganassi M., Matafora V., Parise F., Razzini G., Bachi A., Ferrari S., Molinari S. (2008). Differentiation-dependent lysine 4 acetylation enhances MEF2C binding to DNA in skeletal muscle cells. Nucleic Acids Res..

[52] Liu X., Yang S., Zhao M., Luo M., Yu C.W., Chen C.Y., Tai R., Wu K. (2014). Transcriptional repression by histone deacetylases in plants. Mol. Plant.

[53] Gong X., Tang X., Wiedmann M., Wang X., Peng J., Zheng D., Blair L.A., Marshall J., Mao Z. (2003). Cdk5-mediated inhibition of the protective effects of transcription factor MEF2 in neurotoxicity-induced apoptosis. Neuron.

[54] Kang J., Gocke C.B., Yu H. (2006). Phosphorylation-facilitated sumoylation of MEF2C negatively regulates its transcriptional activity. BMC Biochem..

[55] Xiao Q., Gan Y., Li Y., Fan L., Liu J., Lu P., Liu J., Chen A., Shu G., Yin G. (2021). MEF2A transcriptionally upregulates the expression of ZEB2 and CTNNB1 in colorectal cancer to promote tumor progression. Oncogene.

[56] Minisini M., Di Giorgio E., Kerschbamer E., Dalla E., Faggiani M., Franforte E., Meyer-Almes F.J., Ragno R., Antonini L., Mai A. (2022). Transcriptomic and genomic studies classify NKL54 as a histone deacetylase inhibitor with indirect influence on MEF2-dependent transcription. Nucleic Acids Res..

[57] Ma K., Chan J.K., Zhu G., Wu Z. (2005). Myocyte enhancer factor 2 acetylation by p300 enhances its DNA binding activity, transcriptional activity, and myogenic differentiation. Mol. Cell. Biol..

[58] Gregoire S., Tremblay A.M., Xiao L., Yang Q., Ma K., Nie J., Mao Z., Wu Z., Giguere V., Yang X.J. (2006). Control of MEF2 transcriptional activity by coordinated phosphorylation and sumoylation. J. Biol. Chem..

[59] Jayathilaka N., Han A., Gaffney K.J., Dey R., Jarusiewicz J.A., Noridomi K., Philips M.A., Lei X., He J., Ye J. (2012). Inhibition of the function of class IIa HDACs by blocking their interaction with MEF2. Nucleic Acids Res..

[60] Lu J., McKinsey T.A., Nicol R.L., Olson E.N. (2000). Signal-dependent activation of the MEF2 transcription factor by dissociation from histone deacetylases. Proc. Natl. Acad. Sci. USA.

[61] Wang T., Zhou Y., Bao H., Liu B., Wang M., Wang L., Pan T. (2023). Brusatol enhances MEF2A expression to inhibit RCC progression through the Wnt signalling pathway in renal cell carcinoma. J. Cell Mol. Med..

[62] Ying C.Y., Dominguez-Sola D., Fabi M., Lorenz I.C., Hussein S., Bansal M., Califano A., Pasqualucci L., Basso K., Dalla-Favera R. (2013). MEF2B mutations lead to deregulated expression of the oncogene BCL6 in diffuse large B cell lymphoma. Nat. Immunol..

[63] Zuurbier L., Gutierrez A., Mullighan C.G., Cante-Barrett K., Gevaert A.O., de Rooi J., Li Y., Smits W.K., Buijs-Gladdines J.G., Sonneveld E. (2014). Immature MEF2C-dysregulated T-cell leukemia patients have an early T-cell precursor acute lymphoblastic leukemia gene signature and typically have non-rearranged T-cell receptors. Haematologica.

[64] Galego S., Kauppila L.A., Malho R., Pimentel J., Brito M.A. (2021). Myocyte Enhancer Factor 2C as a New Player in Human Breast Cancer Brain Metastases. Cells.

[65] Cante-Barrett K., Meijer M.T., Cordo V., Hagelaar R., Yang W., Yu J., Smits W.K., Nulle M.E., Jansen J.P., Pieters R. (2022). MEF2C opposes Notch in lymphoid lineage decision and drives leukemia in the thymus. JCI Insight.

[66] Zhang J.J., Zhu Y., Xie K.L., Peng Y.P., Tao J.Q., Tang J., Li Z., Xu Z.K., Dai C.C., Qian Z.Y. (2014). Yin Yang-1 suppresses invasion and metastasis of pancreatic ductal adenocarcinoma by downregulating MMP10 in a MUC4/ErbB2/p38/MEF2C-dependent mechanism. Mol. Cancer.

[67] Zhao L., Zhang P., Galbo P.M., Zhou X., Aryal S., Qiu S., Zhang H., Zhou Y., Li C., Zheng D. (2021). Transcription factor MEF2D is required for the maintenance of MLL-rearranged acute myeloid leukemia. Blood Adv..

[68] Prima V., Hunger S.P. (2007). Cooperative transformation by MEF2D/DAZAP1 and DAZAP1/MEF2D fusion proteins generated by the variant t(1;19) in acute lymphoblastic leukemia. Leukemia.

[69] Li X., Zhang Y., Chai X., Zhou S., Zhang H., He J., Zhou R., Cai L., Chen L., Tao G. (2019). Overexpression of MEF2D contributes to oncogenic malignancy and chemotherapeutic resistance in ovarian carcinoma. Am. J. Cancer Res..

[70] Ma L., Liu J., Liu L., Duan G., Wang Q., Xu Y., Xia F., Shan J., Shen J., Yang Z. (2014). Overexpression of the transcription factor MEF2D in hepatocellular carcinoma sustains malignant character by suppressing G2-M transition genes. Cancer Res..

[71] Bai X.L., Zhang Q., Ye L.Y., Liang F., Sun X., Chen Y., Hu Q.D., Fu Q.H., Su W., Chen Z. (2015). Myocyte enhancer factor 2C regulation of hepatocellular carcinoma via vascular endothelial growth factor and Wnt/beta-catenin signaling. Oncogene.

[72] Pingul B.Y., Huang H., Chen Q., Alikarami F., Zhang Z., Qi J., Bernt K.M., Berger S.L., Cao Z., Shi J. (2022). Dissection of the MEF2D-IRF8 transcriptional circuit dependency in acute myeloid leukemia. iScience.

[73] Zhang M., Truscott J., Davie J. (2013). Loss of MEF2D expression inhibits differentiation and contributes to oncogenesis in rhabdomyosarcoma cells. Mol. Cancer.

[74] Zhu H.X., Shi L., Zhang Y., Zhu Y.C., Bai C.X., Wang X.D., Zhou J.B. (2017). Myocyte enhancer factor 2D provides a cross-talk between chronic inflammation and lung cancer. J. Transl. Med..

[75] Kim Y.J., Oh J., Jung S., Kim C.J., Choi J., Jeon Y.K., Kim H.J., Kim J.W., Suh C.H., Lee Y. (2023). The transcription factor Mef2d regulates B:T synapse-dependent GC-T(FH) differentiation and IL-21-mediated humoral immunity. Sci. Immunol..

[76] Zhang S., Li Z., Zhang L., Xu Z. (2018). MEF2-activated long non-coding RNA PCGEM1 promotes cell proliferation in hormone-refractory prostate cancer through downregulation of miR-148a. Mol. Med. Rep..

[77] Xu K., Zhao Y.C. (2016). MEF2D/Wnt/beta-catenin pathway regulates the proliferation of gastric cancer cells and is regulated by microRNA-19. Tumour Biol..

[78] Nadruz W., Corat M.A., Marin T.M., Guimaraes Pereira G.A., Franchini K.G. (2005). Focal adhesion kinase mediates MEF2 and c-Jun activation by stretch: Role in the activation of the cardiac hypertrophic genetic program. Cardiovasc. Res..

[79] Han T.H., Prywes R. (1995). Regulatory role of MEF2D in serum induction of the c-jun promoter. Mol. Cell. Biol..

[80] McGirt L.Y., Adams C.M., Baerenwald D.A., Zwerner J.P., Zic J.A., Eischen C.M. (2014). miR-223 regulates cell growth and targets proto-oncogenes in mycosis fungoides/cutaneous T-cell lymphoma. J. Investig. Dermatol..

[81] Curtale G. (2018). MiRNAs at the Crossroads between Innate Immunity and Cancer: Focus on Macrophages. Cells.

[82] Cante-Barrett K., Pieters R., Meijerink J.P. (2014). Myocyte enhancer factor 2C in hematopoiesis and leukemia. Oncogene.

[83] Yu W., Huang C., Wang Q., Huang T., Ding Y., Ma C., Ma H., Chen W. (2014). MEF2 transcription factors promotes EMT and invasiveness of hepatocellular carcinoma through TGF-beta1 autoregulation circuitry. Tumour Biol..

[84] Mehta P.B., Robson C.N., Neal D.E., Leung H.Y. (2001). Keratinocyte growth factor activates p38 MAPK to induce stress fibre formation in human prostate DU145 cells. Oncogene.

[85] Ishikawa F., Miyoshi H., Nose K., Shibanuma M. (2010). Transcriptional induction of MMP-10 by TGF-beta, mediated by activation of MEF2A and downregulation of class IIa HDACs. Oncogene.

[86] Sun X., Zhang Y., Xin S., Jin L., Cao Q., Wang H., Wang K., Liu X., Tang C., Li W. (2023). NOTCH3 promotes docetaxel resistance of prostate cancer cells through regulating TUBB3 and MAPK signaling pathway. Cancer Sci..

[87] Cui Y., Miao C., Liu S., Tang J., Zhang J., Bu H., Wang Y., Liang C., Bao M., Hou C. (2021). Clusterin suppresses invasion and metastasis of testicular seminoma by upregulating COL15a1. Mol. Ther. Nucleic Acids.

[88] Sacilotto N., Chouliaras K.M., Nikitenko L.L., Lu Y.W., Fritzsche M., Wallace M.D., Nornes S., Garcia-Moreno F., Payne S., Bridges E. (2016). MEF2 transcription factors are key regulators of sprouting angiogenesis. Genes. Dev..

[89] Li L., Liu J., Wang W., Fu Y., Deng Y., Li X., Liu Z., Pang Y., Xu Y., Yan M. (2023). Cancer stem cells promote lymph nodes metastasis of breast cancer by reprogramming tumor microenvironment. Transl. Oncol..

[90] Garcia A.R., Mendes A., Custodia C., Faria C.C., Barata J.T., Malho R., Figueira I., Brito M.A. (2023). Abrogating Metastatic Properties of Triple-Negative Breast Cancer Cells by EGFR and PI3K Dual Inhibitors. Cancers.

[91] Sereno M., Hasko J., Molnar K., Medina S.J., Reisz Z., Malho R., Videira M., Tiszlavicz L., Booth S.A., Wilhelm I. (2020). Downregulation of circulating miR 802-5p and miR 194-5p and upregulation of brain MEF2C along breast cancer brain metastasization. Mol. Oncol..

[92] Su L., Luo Y., Yang Z., Yang J., Yao C., Cheng F., Shan J., Chen J., Li F., Liu L. (2016). MEF2D Transduces Microenvironment Stimuli to ZEB1 to Promote Epithelial-Mesenchymal Transition and Metastasis in Colorectal Cancer. Cancer Res..

[93] Zhang H., Liu W., Wang Z., Meng L., Wang Y., Yan H., Li L. (2018). MEF2C promotes gefitinib resistance in hepatic cancer cells through regulating MIG6 transcription. Tumori.

[94] Brown F.C., Still E., Koche R.P., Yim C.Y., Takao S., Cifani P., Reed C., Gunasekera S., Ficarro S.B., Romanienko P. (2018). MEF2C Phosphorylation Is Required for Chemotherapy Resistance in Acute Myeloid Leukemia. Cancer Discov..

[95] Chen M.H., Qi B., Cai Q.Q., Sun J.W., Fu L.S., Kang C.L., Fan F., Ma M.Z., Wu X.Z. (2022). LncRNA lncAY is upregulated by sulfatide via Myb/MEF2C acetylation to promote the tumorigenicity of hepatocellular carcinoma cells. Biochim. Biophys. Acta Gene Regul. Mech..

[96] Ma Q., Xu Q., Zhao J., Zhang W., Wang Q., Fang J., Lu Z., Liu J., Ma L. (2021). Coupling HDAC4 with transcriptional factor MEF2D abrogates SPRY4-mediated suppression of ERK activation and elicits hepatocellular carcinoma drug resistance. Cancer Lett..

[97] Suzuki K., Okuno Y., Kawashima N., Muramatsu H., Okuno T., Wang X., Kataoka S., Sekiya Y., Hamada M., Murakami N. (2016). MEF2D-BCL9 Fusion Gene Is Associated With High-Risk Acute B-Cell Precursor Lymphoblastic Leukemia in Adolescents. J. Clin. Oncol..

[98] Liu G., Han J., Profirovic J., Strekalova E., Voyno-Yasenetskaya T.A. (2009). Galpha13 regulates MEF2-dependent gene transcription in endothelial cells: Role in angiogenesis. Angiogenesis.

[99] Wang F., Ding P., Liang X., Ding X., Brandt C.B., Sjostedt E., Zhu J., Bolund S., Zhang L., de Rooij L. (2022). Author Correction: Endothelial cell heterogeneity and microglia regulons revealed by a pig cell landscape at single-cell level. Nat. Commun..

[100] Xiang J., Sun H., Su L., Liu L., Shan J., Shen J., Yang Z., Chen J., Zhong X., Avila M.A. (2017). Myocyte enhancer factor 2D promotes colorectal cancer angiogenesis downstream of hypoxia-inducible factor 1alpha. Cancer Lett..

[101] Wakeland A.K., Soncin F., Moretto-Zita M., Chang C.W., Horii M., Pizzo D., Nelson K.K., Laurent L.C., Parast M.M. (2017). Hypoxia Directs Human Extravillous Trophoblast Differentiation in a Hypoxia-Inducible Factor-Dependent Manner. Am. J. Pathol..

[102] Kong F., Wang L., Lu Z., Zhou J., Ye Q., Hancock W.W., Xiong Y. (2025). MEF2D regulates T-cell function via CD70-CD27 signaling and promotes immune evasion in hepatocellular carcinoma. J. Immunol..

[103] Senapedis W., Gallagher K.M., Figueroa E., Farelli J.D., Lyng R., Hodgson J.G., O’Donnell C.W., Newman J.V., Pacaro M., Siecinski S.K. (2024). Targeted transcriptional downregulation of MYC using epigenomic controllers demonstrates antitumor activity in hepatocellular carcinoma models. Nat. Commun..

[104] Senapedis W., Gallagher K., Figueroa E., Farelli J., O’Donnell C., Newman J., McCauley T. (2022). *P*-307 Modulation of the MYC oncogene using programmable epigenetic mRNA therapeutics as a novel therapy for hepatocellular carcinoma. Ann. Oncol..

[105] Sakamoto K.M., Kim K.B., Kumagai A., Mercurio F., Crews C.M., Deshaies R.J. (2001). Protacs: Chimeric molecules that target proteins to the Skp1-Cullin-F box complex for ubiquitination and degradation. Proc. Natl. Acad. Sci. USA.

[106] Bekes M., Langley D.R., Crews C.M. (2022). PROTAC targeted protein degraders: The past is prologue. Nat. Rev. Drug Discov..

[107] Shao J., Yan Y., Ding D., Wang D., He Y., Pan Y., Yan W., Kharbanda A., Li H.Y., Huang H. (2021). Destruction of DNA-Binding Proteins by Programmable Oligonucleotide PROTAC (O’PROTAC): Effective Targeting of LEF1 and ERG. Adv. Sci. (Weinh.).

[108] Liu J., Chen H., Kaniskan H.U., Xie L., Chen X., Jin J., Wei W. (2021). TF-PROTACs Enable Targeted Degradation of Transcription Factors. J. Am. Chem. Soc..

[109] Hall J., Zhang Z., Bhattacharya S., Wang D., Alcantara M., Liang Y., Swiderski P., Forman S., Kwak L., Vaidehi N. (2024). Oligo-PROTAC strategy for cell-selective and targeted degradation of activated STAT3. Mol. Ther. Nucleic Acids.

[110] Zhang Y., Lin W., Yang Y., Zhu S., Chen Y., Wang H., Teng L. (2024). MEF2D facilitates liver metastasis of gastric cancer cells through directly inducing H1X under IL-13 stimulation. Cancer Lett..

[111] Wang S.Y., Wang Y.X., Guan L.S., Shen A., Huang R.J., Yuan S.Q., Xiao Y.L., Wang L.S., Lei D., Zhao Y. (2025). Construction of a prognostic model for gastric cancer based on immune infiltration and microenvironment, and exploration of MEF2C gene function. BMC Med. Genom..

[112] Xia L., Nie T., Lu F., Huang L., Shi X., Ren D., Lu J., Li X., Xu T., Cui B. (2024). Direct regulation of FNIP1 and FNIP2 by MEF2 sustains MTORC1 activation and tumor progression in pancreatic cancer. Autophagy.

[113] Chopra A., Singh J., Verma D., Kumar R., Bakhshi S., Kumar J., Seth R., Sharma A. (2019). Abstract 5233: MEF2C dysregulation is associated with immature T cell immunophenotype, absence of biallelic deletion of TCR-gamma and inferior survival. Cancer Res..

[114] Torabi A., Fromm J.R., Naresh K.N. (2023). MEF2B is the ideal immunohistochemical marker to highlight neoplastic LP cells in nodular lymphocyte-predominant Hodgkin lymphoma. EJHaem.

